# Inflammation moderates the effects of lifestyle modification on neurocognition among individuals with resistant hypertension

**DOI:** 10.1111/jch.14591

**Published:** 2022-12-20

**Authors:** Forgive Avorgbedor, James A. Blumenthal, Alan Hinderliter, Krista Ingle, Pao‐Hwa Lin, Linda Craighead, Crystal Tyson, William Kraus, Andrew Sherwood, Patrick J. Smith

**Affiliations:** ^1^ School of Nursing University of North Carolina at Greensboro North Carolina USA; ^2^ Department of Psychiatry and Behavioral Sciences Duke University Medical Center Durham North Carolina USA; ^3^ Department of Medicine University of North Carolina at Chapel Hill North Carolina USA; ^4^ Department of Medicine Duke University Medical Center Durham North Carolina USA; ^5^ Department of Psychology Emory University Atlanta Georgia USA

**Keywords:** cognitive function, inflammation, lifestyle modification, resistant hypertension

## Abstract

Individuals with resistant hypertension (RH) have the greatest risk of cerebrovascular disease and cognitive impairment among individuals with hypertension. Elevated levels of pro‐inflammatory cytokines may represent a critical yet unexamined factor influencing the impact of healthy lifestyle changes on cognitive function. We explored the influence of inflammation on changes in cognition following lifestyle modification among individuals with RH participating in the TRIUMPH clinical trial. One hundred forty participants with RH completed a battery of neurocognitive tests along with the inflammatory marker C‐reactive protein (hsCRP) and were subsequently randomized to an intensive 4‐month lifestyle modification intervention or to education and physician advice control. Results indicated that the effects of lifestyle modification on Executive Function and Learning were moderated by pre‐intervention hsCRP levels (*P* = .049), with treatment efficacy increasing across levels of baseline inflammation levels (low: *d* = 0.12; mild: *d* = 0.43; moderate: *d* = 0.81). We conclude that inflammatory profiles may help identify individuals more likely to improve executive functioning resulting from lifestyle modification.

## 1 INTRODUCTION

Hypertension is one of the primary causes of cardiovascular disease, stroke, Alzheimer's Disease, and Alzheimer's Disease and related dementias (AD/ADRD).^1^ Among individuals with hypertension, those with resistant hypertension (RH) appear to have the greatest risk of cerebrovascular disease and associated cognitive impairment.^2^ Although the deleterious effects of RH on cognitive function are likely multifactorial, emerging evidence suggests that elevated levels of pro‐inflammatory cytokines may be a critical, central mechanism by which RH worsens cognition.^3^, ^4^ Indeed, recent reviews have emphasized that hypertension, CVD risk factors, and age‐related cognitive decline all share common underlying mechanisms of risk for cognitive decline, with inflammation serving as the most likely convergent mechanistic pathway.^5^ Understanding the overlapping influences of hypertension and inflammation are particularly important as recent consensus guidelines have advocated for targeted lifestyle modification as a preventive strategy to mitigate ADRD risk among vulnerable adults, with precision medicine frameworks emphasizing the use of elevated inflammatory cytokines to identify vulnerable middle‐aged adults for lifestyle changes.^5^

Lifestyle modification, including exercise and dietary modification, is an established approach to improve both hypertension and cognitive function. The TRIUMPH clinical trial^6^ demonstrated that a comprehensive exercise and dietary program can improve blood pressure, weight, and CVD biomarkers,^6^ as well as improving neurocognitive function.^7^ Emerging evidence suggests that individual differences in CVD risk factors and background characteristics may moderate improvements neurocognition, particularly in the domain of executive function.^7^, ^8^ Despite these reported individual differences and the potentially integral role of inflammation, to our knowledge no studies have examined inflammation as a potential moderator of improved cognitive function following lifestyle modification. Therefore, this brief report aimed to investigate the potential influence of individual differences in pre‐treatment inflammatory profiles on changes in cognition following lifestyle modification among RH participants in the TRIUMPH clinical trial.^6^

## 2 METHODS

The methodology for the present report has been previously published.^6^, ^7^ Briefly, the TRIUMPH study was a randomized clinical trial (RCT) designed to evaluate whether intensive lifestyle modification could reduce BP and improve CVD biomarker outcomes among 140 individuals with RH relative to an education and physician advice control condition.^6^ Details of the primary trial results and neurocognitive outcomes have been reported previously (NCT03001427 and NCT03001427).^6^, ^7^ One hundred forty patients with RH were randomized with 2:1 allocation to either a 4‐month Center‐based Lifestyle intervention (C‐LIFE) or Standardized Education and Physician Advice (SEPA).

Neurocognitive function was assessed by a battery of standard neurocognitive tests, which are detailed in the primary paper.^7^ The primary endpoint of the TRIUMPH cognitive study was Executive Function and Learning, with Memory and Processing Speed serving as secondary outcomes. Participants were also screened for dementia using the Montreal Cognitive Assessment test (MOCA).

Inflammation was assessed sing high‐sensitivity C‐reactive protein (hsCRP), quantified by Lab Corp using commercial enzyme‐linked immunosorbent assay kits (R&D Systems). Values exceeding 10 mg/dl were truncated at 10 mg/dL, consistent with contemporary analytical guidelines.

### 2.1 Data analysis

All analyses were conducted using SAS 9.4 (Cary, North Carolina) and R 4.1.3 (https://cran.r‐project.org/). General linear models were used to examine the potential moderating effect of baseline inflammation on 1) the relationship between vascular risk factors and cognitive function at baseline and 2) treatment‐related changes in cognitive function. Within each model, adjustment variables included age, education, biological sex, race (minority vs. non‐minority), Framingham Stroke Risk Profile (FSRP) score, creatinine, and hsCRP. For our analyses of treatment‐related changes in cognitive function, we also controlled for the baseline level of the respective cognitive outcome and group assignment. In order to test for potential moderation by baseline inflammatory markers, we tested for interactions between hsCRP and group assignment for potential treatment group moderation. We also incorporated follow‐up data collected at one‐year collected in a subset of participants prior to the onset of the COVID‐19 pandemic lockdown (*n* = 91). In order to account for missing data, multiple imputations were used (PROC MI) with 50 imputations. For analyses of follow‐up data, we used a repeated measures mixed model with an unstructured covariance matrix and the same covariates as our primary analyses above.

Exploratory analyses were also conducted to examine the association between changes in putative causal treatment mechanisms (aerobic fitness, weight loss, dietary changes, and BP) and cognitive outcomes for the purposes of hypothesis generation. Model assumptions regarding linearity, independence, and normality of residuals were assessed and found to be acceptable prior to analysis. For explanatory purposes, we also characterized the sample by baseline levels of hsCRP. To do so, we utilized previously published hsCRP clinical thresholds from baseline assessments, with hsCRP levels of ≤2.0 mg/L, 2–5 mg/L, and ≥5.0 mg/L used to delineate normal, mildly elevated, and moderately elevated CRP groups. Following convention, hsCRP levels > 10 mg/L were truncated for the purposes of analyses, and hsCRP was log‐transformed within regression models in order to preserve the normality of residuals.

## 3 RESULTS

Examination of baseline hsCRP levels revealed that 49 individuals (35%) exhibited normal levels, 51 individuals (36%) exhibited mildly elevated levels, and 40 individuals (29%) exhibited moderately elevated levels. There were no baseline differences in hsCRP levels between treatment groups (*P* = .817). Examination of Table 1 shows that higher hsCRP levels were associated with greater obesity (*r* = 0.45, *P* < .001) and HOMA‐IR (*r* = 0.31, *P* < .001), and younger age (*r* = −0.24, *P* = .004).

**TABLE 1:** Baseline demographic, clinical, and neurocognitive characteristics of the sample by CRP levels and treatment group. Scores within cognitive domains reflect global, mean‐rank scores where higher performance is consistent with better performance relative to other trial participants

	**Low CRP** (*n* = 49)		**Mildly elevated CRP** (*n* = 51)		**Moderately elevated CRP** (*n* = 40)	
**Variable**	**C‐LIFE** (*n* = 35)	**SEPA** (*n* = 14)	**C‐LIFE** (*n* = 29)	**SEPA** (*n* = 22)	**C‐LIFE** (*n* = 26)	**SEPA** (*n* = 14)
**Demographic and background characteristics**						
Age, years	63.3 (9.3)	62.6 (9.7)	62.7 (8.4)	64.5 (9.4)	59.9 (8.2)	61.7 (9.2)
Male, *n* (%)	20 (67%)	10 (33%)	17(65%)	9 (35%)	10 (59%)	7 (41%)
Minority, *n* (%)	18 (58%)	13 (42%)	16 (53%)	14 (47%)	17 (59%)	12 (41%)
Duration of hypertension, years	23.2 (12.5)	25.5 (12.2)	20.3 (12.0)	18.1 (8.1)	22.9 (13.4)	25.7 (10.6)
**Clinical characteristics**						
Clinic systolic blood pressure, mmHg	136.6 (9.7)	138.7 (10.8)	141.1 (11.2)	139.2 (8.5)	138.8 (10.3)	143.9 (10.1)
Clinic diastolic blood pressure, mmHg	77.6 (10.2)	81.0 (10.0)	79.2 (9.2)	78.4 (8.5)	80.1 (8.2)	80.9 (6.7)
Body mass index, kg/m2**	32.4 (5.0)	35.7 (5.3)	36.8 (5.1)	35.1 (5.0)	39.5 (5.8)	38.1 (5.2)
Waist to hip ratio, %	98.7 (6.0)	98.9 (8.4)	98.2 (6.3)	96.9 (6.1)	97.1 (6.8)	99.6 (5.6)
Homeostatic model of insulin resistance, U**	3.6 (1.9)	3.8 (2.2)	5.2 (2.8)	5.2 (3.9)	5.8 (2.7)	5.1 (4.0)
Framingham stroke risk profile score	10.4 (3.4)	9.6 (3.2)	9.9 (3.7)	11.3 (3.6)	9.5 (3.2)	10.6 (2.5)
Atherosclerotic risk profile score	13.5 (9.0)	10.6 (7.6)	14.9 (8.3)	14.1 (8.2)	11.8 (6.4)	14.4 (9.7)
Creatinine, mg/dl	84.7 (31.7)	99.5 (48.7)	77.5 (30.8)	86.7 (50.0)	79.0 (38.1)	85.1 (32.3)
**Cognitive characteristics**						
Montreal cognitive assessment score	25.5 (2.5)	25.5 (2.0)	25.9 (2.2)	25.5 (2.6)	25.5 (2.6)	23.5 (4.0)
Executive function/learning score, mean rank	68.9 (20.8)	68.3 (26.4)	69.7 (22.7)	65.5 (21.6)	74.7 (15.3)	67.9 (21.6)
Processing speed score, mean rank	74.7 (31.5)	63.1 (33.7)	74.3 (27.1)	68.9 (29.7)	69.4 (23.2)	60.2 (35.1)
Memory score, mean rank	66.8 (29.2)	61.6 (35.0)	70.3 (27.6)	70.8 (30.8)	77.4 (26.1)	67.2 (32.8)

Omnibus test for differences between CRP groups: **P < .01; *P < .05.

### 3.1 Baseline inflammation and treatment‐related cognitive changes

**FIGURE 1:**
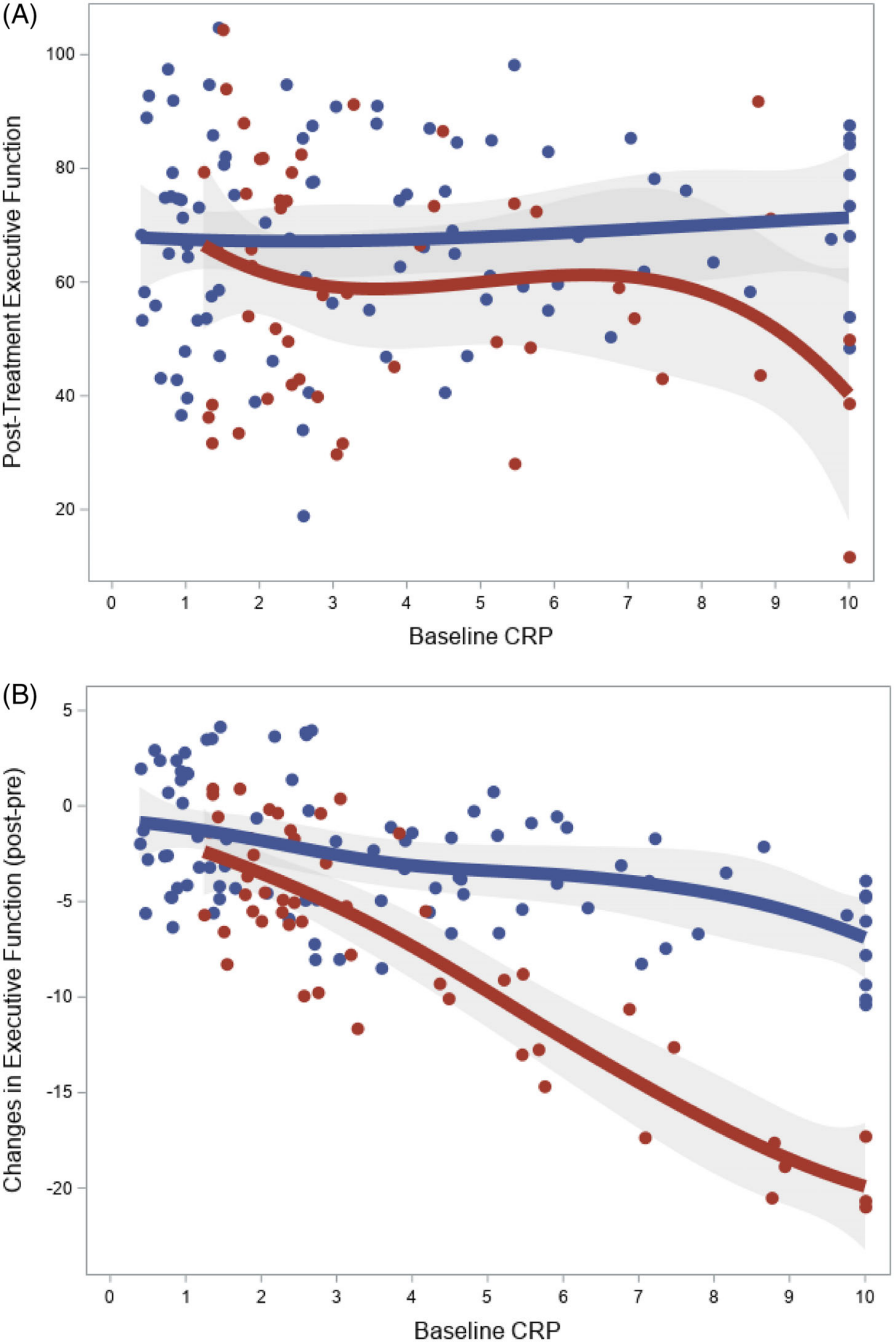
Treatment group differences across levels of baseline inflammation from adjusted regression models (C‐LIFE = blue; SEPA = red). As shown, post‐treatment Executive Function performance (A) and changes in Executive Function (B) worsened in the SEPA group at higher levels of baseline CRP. In contrast, changes were relatively consistent in C‐LIFE across baseline CRP levels

Examination of treatment‐related changes in cognitive function revealed that the effects of treatment on Executive Function and Learning were moderated by baseline hsCRP levels (*P* = .049); there was no evidence of moderation for Processing Speed (*P* = .897) or Memory (*P* = .936). Explanatory analyses revealed that treatment group differences were minimal among those with low baseline CRP levels and that treatment group differences were greater among those participants with higher baseline CRP levels. Specifically, comparison of post‐treatment performance between those in C‐LIFE vs. SEPA were smallest for low hsCRP (*d* = 0.12; 67.5 [63.5, 71.5] vs. 69.1 [61.7, 76.4]), intermediate for mildly elevated hsCRP (*d* = 0.43; 67.9 [63.4, 72.3] vs. 62.3 [56.6, 67.9]), and greatest for moderately elevated hsCRP (*d* = 0.81; 64.2 [59.5, 68.9] vs. 53.6 [46.4, 60.9]). Using demographically‐corrected normative data, among individuals in the higher hsCRP group, these differences corresponded to improvements of 1.5 (−0.2, 3.1) and 0.2 (−2.4, 2.8) in the C‐LIFE and SEPA groups, respectively. In addition, we observed a non‐linear association between hsCRP and changes in Executive Function and Learning across groups (*P* = .019 for restrictive cubic spline test), such that group effects diverged at approximate hsCRP levels of 3.0 and 7.0 mg/L, respectively (Figure 1). Examination of follow‐up neurocognitive outcomes collected at one‐year following randomization were also examined. We found that hsCRP continued to moderate the effects of treatment when one‐year follow‐up data were incorporated as an additional repeated measures outcome (P = .030). Results for individuals with low, mild, and moderate hsCRP levels demonstrated better performance in C‐LIFE vs. SEPA at both T2 (C‐LIFE: 73.3 [69.1, 77.5], 73.4 [68.8, 78.1], and 69.8 [64.8, 74.7] vs. SEPA: 74.8 [67.1, 82.4], 67.8 [61.8, 73.7], and 58.2 [50.6, 65.9]) and T3 (C‐LIFE: 73.1 [68.1, 78.1], 68.5 [62.9, 74.1], and 68.8 [62.5, 75.1], vs. SEPA: 75.4 [65.8, 85.0], 69.6 [62.6, 76.6], and 58.2 [50.2, 66.2]).

Exploratory analyses of putative treatment changes revealed that the moderating effects of treatment were paralleled by moderating effects of aerobic fitness improvements (*P* = .045), such that the effects of improved peak oxygen consumption on Executive Function and Learning were observed among individuals with moderately elevated baseline hsCRP (*r* = 0.29, *P* = .038), but not among those with mild (*r* = 0.04, *P* = .619), or low levels (*r* = 0.03, *P* = .775). No moderating effects were observed for weight loss, dietary changes, or BP reductions. We also found no evidence that treatment differentially improved hsCRP levels, or that reductions in hsCRP from pre‐to‐post treatment associated with improvements in Executive Function and Learning (*B* = −0.10, *P* = .192).

## 4 DISCUSSION

The present findings extend prior work demonstrating that basal levels of elevated peripheral inflammation may represent an intermediate phenotype of risk for cognitive decline.^9^, ^10^ We found that individuals with higher levels of hsCRP at baseline demonstrated greater improvements in Executive Function/Learning following participation in an intensive lifestyle intervention. These findings may suggest that inflammatory profiles may help identify individuals more likely to benefit cognitively from lifestyle modification using exercise and dietary modification.

Prior epidemiological studies have suggested that elevated inflammatory profiles may interact with comorbid cardiometabolic risk factors to adversely affect cognitive functioning,^11^ although no prior randomized trials have examined such individual differences. CVD risk factors, including hypertension and obesity, have consistently been linked to cognitive decline through an array of interrelated mechanisms including impaired insulin sensitivity and elevated inflammation.^12^, ^13^ Because of the overlapping influences of CVD risk, obesity, and metabolic dysfunction on inflammation, emerging data have suggested that elevated inflammatory markers among individuals with CVD risk factors may represent a viable intermediate phenotype of ADRD risk, identifying adults with a higher likelihood of ADRD through the convergent influence of multiple risk pathways.^9^, ^10^ Among individuals with type‐2 diabetes, for example, greater plasma hsCRP concentrations continue to predictive of subsequent cognitive decline,^14^ suggesting that a pro‐inflammatory state confers ADRD risk beyond the influence of metabolic dysfunction. Similarly, prior systematic reviews have suggested that the linkages between obesity and impaired executive function are likely indirect or amplified by comorbid systemic risk factors, suggesting elevated inflammation may represent a downstream, convergent pathway of risk driven by an array of CVD risk markers.^15^ This is consistent with findings in the present study among sedentary adults with RH, as neither baseline obesity (*P* = .656) nor insulin sensitivity (*P* = .444) moderated treatment changes on Executive Function, despite both associating with baseline hsCRP concentrations. Our findings therefore extend prior research by demonstrating that elevated inflammation in the presence of CVD risk factors may impact cognitive changes following lifestyle modification, providing initial evidence to refine targeted treatment approaches.

### 4.1 Limitations

First, the intervention was relatively brief and future studies will need to examine whether individual treatment differences based on inflammatory profiles persisted over follow‐up. Second, our assessment of inflammation was limited to hsCRP, whereas future studies should collect a richer array of inflammatory markers. However, we note that when inflammatory data from a subset of participants who also had tumor necrosis factor‐alpha levels collected were integrated with hsCRP, our observation that baseline inflammation moderated the effects of treatment persisted (*P* = .039). Third, our assessment of inflammatory markers was limited to peripheral serum markers and may therefore not reflect inflammation within the central nervous system. Finally, our 2:1 randomization resulted in a 1.8:1 ratio of C‐LIFE to SEPA participants, despite demonstrating very few baseline group differences.^6^

## 5 CONCLUSION

The findings in this report extend to previous studies on the role of inflammation in cognitive dysfunction by demonstrating that the beneficial effects of lifestyle modification may be greater among individuals with greater levels of inflammation. These findings, if replicated, may help inform targeted treatments to reduce ADRD among middle‐aged and older adults with CVD risk factors.

## CONFLICT OF INTEREST

None.
